# Factors contributing to the promotion of moral competence in nursing

**DOI:** 10.1177/09697330241235305

**Published:** 2024-03-20

**Authors:** Johanna Wiisak, Minna Stolt, Michael Igoumenidis, Stefania Chiappinotto, Chris Gastmans, Brian Keogh, Evelyne Mertens, Alvisa Palese, Evridiki Papastavrou, Catherine Mc Cabe, Riitta Suhonen, On behalf of the PROMOCON consortium

**Affiliations:** 8058 University of Turku; 8058 University of Turku; Wellbeing Services County of Satakunta; 37795 University of Patras; 9316 University of Udine; 26657 KU Leuven; 8809 Trinity College Dublin; 26657 KU Leuven; 9316 University of Udine; Cyprus University of Technology; Cyprus Nurses and Midwifes Association; 8809 Trinity College Dublin; 8058 University of Turku; 60652 Turku University Hospital; Wellbeing Services County of Southwest Finland

**Keywords:** Moral competence, nursing, nursing students, qualified nurses, integrative review, ethical competence

## Abstract

Ethics is a foundational competency in healthcare inherent in everyday nursing practice. Therefore, the promotion of qualified nurses’ and nursing students’ moral competence is essential to ensure ethically high-quality and sustainable healthcare. The aim of this integrative literature review is to identify the factors contributing to the promotion of qualified nurses’ and nursing students’ moral competence. The review has been registered in PROSPERO (CRD42023386947) and reported according to the PRISMA guideline. Focusing on qualified nurses’ and nursing students’ moral competence, a literature search was undertaken in January 2023 in six scientific databases: CINAHL, Cochrane Library, PsycINFO, PubMed Medline, Scopus and Web of Science. Empirical studies written in English without time limitation were eligible for inclusion. A total of 29 full texts were retrieved and included out of 5233 citations. Quality appraisal was employed using Joanna Briggs Institute checklists and the Mixed Method Appraisal Tool. Data were analysed using inductive content analysis. Research about the factors contributing to the promotion of qualified nurses’ and nursing students’ moral competence is limited and mainly explored using descriptive research designs. The contributing factors were identified as comprising two main categories: (1) human factors, consisting of four categories: individual, social, managerial and professional factors, and ten sub-categories; and (2) structural factors, consisting of four categories: educational, environmental, organisational and societal factors, and eight sub-categories. This review provides knowledge about the factors contributing to the promotion of qualified nurses’ and nursing students’ moral competence for the use of researchers, nurse educators, managers, organisations and policymakers. More research about the contributing factors is needed using complex intervention, implementation and multiple methods designs to ensure ethically sustainable healthcare.

## Introduction

Ethics is a foundational competency in healthcare^1^ and is inherent in all nursing practices.^2^ Healthcare professionals demonstrate their ethical competence in providing high-quality care to patients.^3^ Constantly changing society and healthcare environments with ever-growing demands for ethically sustainable care require nurses’ and nursing students’ moral competence to evolve continuously. This became more evident during the COVID-19 pandemic which required nurses to make difficult ethical decisions,^4,5^ and with the phenomenon of missed care that requires nurses to prioritise scarce resources.^6^ Therefore, it is important to develop a support system to promote and sustain nurses’ and nursing students’ moral competence.

## Background

The terms moral competence and ethical competence have been used interchangeably in the literature.^7^ Moral competence has been defined as ‘the ability or capacity of persons to recognise their feelings as they influence what is good or bad in particular situations, and then to reflect on these feelings, to make their decision, and to act in ways that bring about the highest level of benefit for patients’.^8^ (p. 586). Ethical competence in healthcare, in turn, is defined as ‘a personal capacity including ethical awareness, courage, willingness and skills in decision-making and ethical action’.^9^ (p. 410). As moral competence and ethical competence have been used synonymously, in this review, the concept of moral competence is used and defined in terms of perceptions (seeing), knowledge (knowing), reflection, deliberation and acting as a professional caregiver.^10^

There is a wealth of literature on what constitutes nurses’ moral competence,^3,7,9^ as well as the level of their moral competence^11^ and the variables associated with it, such as ethics education,^12,13^ and nurses’ ethical reasoning and behaviour.^14^ However, the literature on factors that contribute to the promotion of qualified nurses’ and nursing students’ moral competence is limited. Factors have been considered by the research team in this study context as those elements facilitating, contributing or leading to a specific outcome, as the promotion of moral competence among nurses and nursing students. The pedagogical approaches in teaching or learning ethics in undergraduate nursing education have been found to be limited in terms of what should be taught about ethics, and how and by whom it should be taught.^15^ There is also great variation and diversity globally, with some countries offering ethics as stand-alone courses while in others, it is integrated into other subject areas. Similarly, the delivery of ethics education in nursing education varies with practices like clinical and didactic courses including discussions, simulation, case-based learning, problem-based techniques, role play and analysing ethical issues.^16^ In view of the increasing complexity of care and the accompanying emergence of new ethical challenges, there is a need to re-examine the content of nursing curricula to enhance the promotion of morally competent nurses as well as teaching practices that are more adaptive to the changing learning needs.

Factors that support and contribute to the promotion of moral competence have been identified not only in education but also in clinical settings. The main aims of support are to improve ethical decision-making and action in the clinical environment, to prevent, mitigate or reduce the ethical burden, supervise policymaking and provide guidelines, education and consultation.^17^ The ethical competence of healthcare professionals in undergraduate, continuing and clinical education can be promoted through various interventions, especially educational ones.^18^ However, an integrative summary of the factors that may contribute to the promotion of moral competence was not identified from the literature.

The importance of ethics in healthcare and the requirement of moral competence from nursing professionals in providing high-quality care with respect to human and patients’ rights have been acknowledged in the literature.^3,4,9^ In addition, ethics education in nursing curricula and the best practices varies globally.^16^ Therefore, this review was conducted to identify the factors that can contribute to the promotion of moral competence of qualified nurses and nursing students not only in educational institutions but also in healthcare organisations.

## Aim

The aim of this review was to identify the factors contributing to the promotion of qualified nurses’ and nursing students’ moral competence.

## Methods

An integrative review method was used and its five steps followed according to the methodological model of Whittemore and Knafl,^19^ namely, (a) problem-identification, which ensures that both the research question and purpose are appropriately formulated; (b) research strategy and literature search; (c) methodological quality assessment, (d) data analysis; and (e) presentation and synthesis of findings. The review protocol has been registered in the International Prospective Register of Systematic Reviews (CRD42023386947). The Preferred Reporting Items for Systematic Reviews and Meta-analyses (PRISMA)^20^ was followed.

### Search strategy

A literature search regarding factors contributing to qualified nurses’ and nursing students’ moral competence was carried out to identify relevant scientific research articles. The searches were undertaken on 2^nd^–4^th^ January 2023 from the earliest content in six scientific databases: CINAHL, Cochrane Library, PsycINFO, PubMed Medline, Scopus and Web of Science. The search terms and strategies were developed in collaboration with a health and medical science library informatics expert. The following terms were used with the Boolean operators AND or OR: (nurse OR nursing OR nursing student OR student nurse) AND (moral competence OR ethical competence OR ethical sensitivity OR ethical decision-making OR ethical knowledge OR ethical behaviour OR ethical behaviour OR ethical reflection OR ethical reasoning OR moral courage OR moral care). The keywords were applied in all databases and MeSH terms were also used when appropriate. In addition, the reference lists of the included articles and relevant reviews were screened; however, this did not produce any new articles to the review. The Zotero software was used to manage references.^21^

### Inclusion and exclusion criteria

The studies were included if they focused on (1) the moral competence of (2) qualified nurses (registered nurses, practical nurses, Advanced Practice Nurses) or nursing students, including articles that also covered other healthcare professionals in addition to them, (3) identify the factors contributing to the promotion of moral competence, (4) peer-reviewed empirical articles (including all research methods), written in (5) English language and with (6) abstract available. The studies were excluded if they dealt with (1) the moral competence of manager-level nurses, nurse aids, nurse assistants, or solely of other healthcare professionals, and if the articles were (2) theoretical articles, literature reviews, books, dissertations, reports, editorials, opinions, discussion papers or grey literature.

### Quality appraisal

Quality appraisal was employed using the Joanna Briggs Institute Critical Appraisal Checklists for Qualitative Research in twelve studies, for Analytical Cross-Sectional Studies in eleven studies, and for Quasi-Experimental Studies in five studies.^22^ However, for one mixed-method study the Mixed Method Appraisal Tool (MMAT)^23^ was used instead, as no Joanna Briggs Institute (JBI) checklist existed for this purpose. The ranking of the JBI checklist items included Yes, No, Unclear and Not applicable. The quality of the included articles was rated as moderate (*n* = 10) or high quality (*n* = 18). The mixed-method article was rated as low quality. However, as suggested in the literature, none of them were excluded based on quality appraisal.^23^ The quality appraisal of each article was carried out by two researchers. A total of eight researchers (JW, MS, SC, CG, BK, EM, EP, RS) participated and discrepancies were resolved among three researchers (JW, MS, RS).

### Data analysis

A data extraction template was constructed for the review and data from the included articles were extracted and inserted into the template to describe the existing literature. The information included the author(s), year, country of origin, aim(s), study design/methods, setting/sample, factors contributing to moral competence and reported outcomes. Data were analysed by four researchers (JW, MS, MI, RS) using inductive content analysis.^24^ First, the articles were scrutinized by reading them thoroughly several times. During familiarisation, notes were made on the manifest content. Second, the unit of analysis was selected as the original expressions of the authors of the articles that were considered relevant to respond to the aim of the review. These were drawn into table worksheet as meaning units. Third, meaning units were further reduced into condensed meaning units. Fourth, condensed meaning units were abstracted and interpreted by comparing them and notes for differences and similarities and sorted into sixteen sub-categories and further into eight categories. Finally, two main categories that unified the content in the categories were formulated (Table 1).

**Table 1.** Example of data analysis.

Meaning unit	Condensed meaning unit	Sub-category	Category	Main category
‘The learning experience of being situated within the scenario and being able to explore their own feelings in connection with what was happening’ (Oddvang et al. 2021).	Explore one’s own feelings	Self-reflection	Individual factors	Human factors
‘Strong will to face difficult situations’ (Asahara et al. 2014, Göl & Arkan 2022).	Strong will	Character strength		
‘Moral transition: At this stage, students are motivated to accomplish these skills, which they see modelled by both their educators and their peers’ (Ranjbar et al. 2018).	Seeing modelled by both their educators and their peers	A professional role model	Social factors	
‘Discussing the ethical problems of each patient’ (Borhani et al. 2010)	Discussing the ethical problems	Mutual interaction		
‘An interactive situational e-learning system, integrating traditional face to face teaching with an interactive multimedia online system’. (Chao et al. 2017)	An interactive situational e-learning system	Teaching methods	Educational factors	Structural factors
‘The practice scenario… facing the problem, assuming responsibility for solving it and taking actions grounded in values and knowledge’ (Schaefer & Junges 2014).	Face to face teaching			
	Hypothetical scenarios	Content of teaching		
‘Multidisciplinary discussion of ethical issues’ (Maluwa et al. 2021).	Lack of ethical guidelines, protocols or frameworks	Ethics management strategies	Organisational factors	
‘Availability of resources including guidelines, rules and protocols as part of material resources’ (Maluwa et al. 2021).	Availability of resources	Resources of the organization		

## Findings

### Studies retrieved

**Figure 1.**
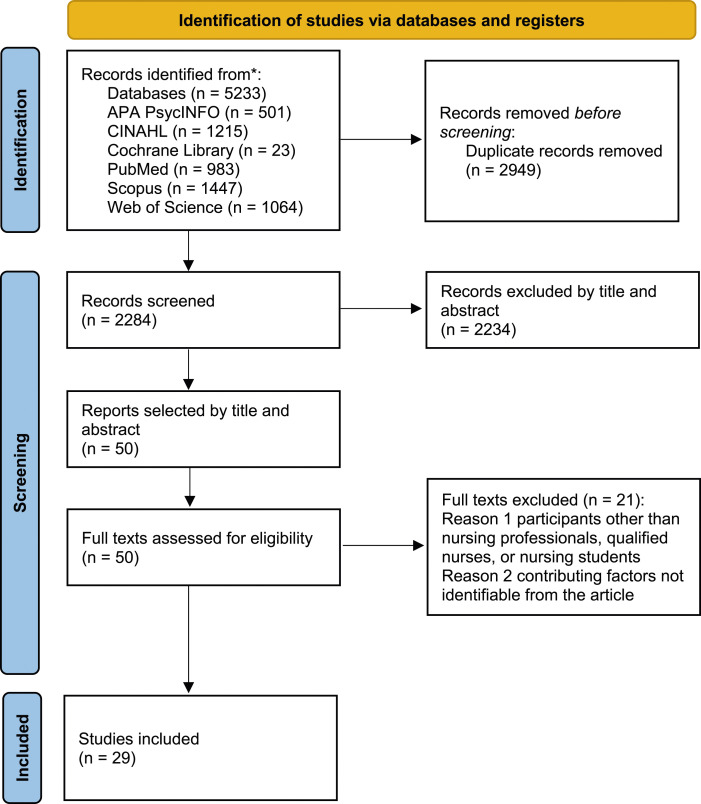
The PRISMA flow diagram, 2020 statement: An updated guideline for reporting systematic reviews.^20^

The studies were retrieved according to the four stages of the PRISMA^20^ flowchart (Figure 1). At the first stage, 5233 records were identified from six databases; 2949 duplicates were removed. The remaining 2284 records were then screened by their titles and abstracts. Following this, 50 full text reports remained, which were screened for eligibility. Twenty-one reports were excluded. In the last stage, 29 studies were included in the review.

### Characteristics of the studies

The studies (*n* = 29) were published between 2007 and 2022, the majority (*n* = 17) between 2010 and 2019, only one study before 2010,^25^ and the rest of the studies (*n* = 11) in 2020 or later (Table 2). Four studies were carried out in Finland^26–29^ and three in Iran.^30–32^ Two studies were conducted in each of the following countries Brazil,^33,34^ Norway,^35,36^ Japan,^37,38^ Portugal,^39,40^ Sweden^25,41^ and Turkey.^42,43^ One study was conducted in each of the following countries: Belgium,^44^ Canada,^45^ Czech Republic,^46^ Israel,^47^ Malawi,^48^ Slovenia,^49^ South Korea,^50^ Spain,^51^ Taiwan^52^ and the Netherlands.^53^

**Table 2.** Studies (*n* = 29) about moral competence included in the review according to study design.

Authors, year, country	Aim	Study design/methods	Setting/sample	Moral competence contributing factors	Reported outcomes	Quality appraisal points^a^
**Qualitative studies**						Qualitative (ten items)
Borhani et al., 2010, Iran	Survey the experience of nursing students with respect to the role of instructors in the promotion of professional ethics.	A qualitative study with in-depth interviews	Nursing students (*n* = 15)	Experiences in the role of the instructor in the promotion of professional ethics.	Two main themes and seven sub-categories: (1) the effective professional role model encompassing (a) individual characteristics and beliefs, (b) clinical skills, (c) professional commitment of role model (2) creative learning facilitates, (d) critical thinking and decision-making, (e) providing supportive learning conditions, (f) providing proper space for sharing knowledge, (g) evaluation and creative feedback.	7/10
Díaz Agea et al. 2018, Spain	To analyse students’ perceptions of the process of learning ethics through simulations and to describe the underlying frames that inform the decision-making process of nursing students.	A qualitative study approach with simulated scenarios video filmed	Fourth-year nursing students (*n* = 30)	Perceptions on learning and acquisition of ethical competence through simulations.	The students’ perspective regarding their learning and acquisition of ethical competence through simulations was positive. Six simulated scenarios and ethical competencies related to (1) autonomy, (2) ethical reaction to suspected abuse, (3) suicide from the ethical point of view, protection of life vs. autonomy, informed consent, (4) ethical subjects related to CPR, (5) therapeutic relentlessness, (6) autonomy.	7/10
Enderle et al., 2018, Brazil	To identify strategies and spaces used by professors to promote the development of the moral competence of nursing undergraduate students.	A qualitative study with semi-structured interviews	Nursing Professors (*n* = 20)	Teaching strategies and spaces that promote the development of moral competence.	Three categories: (1) Active methodologies as strategies for the development of moral competence, (2) Knowledge and development of clinical reasoning as motivating spaces of moral competence, (3) Attitude of professors as a strategy for dialogue, empathy, recovery of moral values and development of caring skills	7/10
					Intermediate categories: (a) teaching strategies, (b) active methodologies, (c) study dynamics, (d) knowledge, (e) clinical reasoning, (f) moral competence, (g) attitude of professors, (h) dialogue, and (i) moral values	
Hemberg & Hemberg, 2020, Finland	To investigate healthcare professionals’ views on ethical competence in a student healthcare context.	A qualitative design and a hermeneutical approach with interviews	Nurses (*n* = 9)	Views on ethical competence.	The main theme: safeguarding the vulnerability of the other. Four sub-themes: (a) using sensitivity to establish a trustful relationship, (b) acting in an objective and flexible manner, (c) using a reflective process in decision-making, (d) maintaining confidentiality and honesty.	8/10
			Other (*n* = 1)			
Höglund et al., 2010, Sweden	To describe and explore ethical dilemmas that Swedish research nurses experience in their day-to day work.	A qualitative study with in-depth interviews	Research nurses (*n* = 6)	Experiences on ethical dilemmas.	Three themes: (1) ethical dilemmas, (2) ethical reasoning, (3) attitudes towards research nurses and their work.	8/10
					Sub-categories: (a) research interests versus the patient’s interests, (b) feeling responsible, (c) ethical disagreements with the PI, (d) conflicting roles, (e) working patient-centred, (f) an ‘invisible’ profession and (g) not encouraged to develop ethical competence.	
Maluwa et al. 2021, Malawi	To explore the factors that affect ethical competence.	A qualitative exploratory design with in-depth interviews and focus groups	Registered Nurses and Registered Nurse Midwives (*n* = 44)	Perceptions on the factors affecting ethical competence.	Main theme: Systems influencing ethical competence among clinical nurses in Malawi.	7/10
			Other (*n* = 29)		Sub-themes: (1) Perceptions of clinical nurses on nurse managers’ roles in influencing ethical competence, (2) Nurses’ perceptions on the nurse leaders’ attributes that influence ethical competence.	
Oddvang et al., 2021, Norway	To consider the potential role of simulation in helping nursing students build the ethical awareness and competence that are such important aspects of person-centred care.	A qualitative, explorative design, using focus group interviews.	2-year nursing students (*n* = 9)	Educational intervention of a 2-week simulation programme with scenarios.	Experience gained through participation and acting in simulations. The shared experience was a good starting point for guided reflection on ethical and tacit knowledge. The acquired experience led to knowledge transferable to similar situations in clinical practice.	8/10
					2-week simulation programme with scenarios and phases: briefing, action and debriefing. Ethical focus: (1) Ethics of duty, consequence, autonomous choices, (2) Ethics of proximity, the four principles, (3) Autonomy, paternalism	
Peter et al., 2015, Canada	To explore nurses’ moral competence related to fostering hope in patients and their families within the context of aggressive technological care.	A critical qualitative approach with interviews	Graduate nursing students (*n* = 15)	Experiences of moral distress while providing care.	Mediating the tension between providing false hope and destroying hope within biomedicine. Three sub-themes: Reimagining hopeful possibilities; Exercising caution within the social–moral space of nursing; Maintaining nurses’ own hope.	9/10
Ranjbar et al., 2018, Iran	To explore the moral development process in nursing students.	A constructivist grounded theory, interviews	Nursing students (*n* = 19)	Experiences of the changes in practical skills, moral capabilities and competencies.	Students experience changes to both their practical skills and their moral capabilities and competencies. Three main steps: moral transition, moral reconstruction, and moral internalization. Techno-scientific competence, biomedical competence, and nursing competence are developed in each step, respectively.	9/10
			Nursing instructors (*n* = 3)			
Schaefer & Junges, 2013, Brazil	To understand the perceptions of primary healthcare services nurses on the construction process of ethical competence.	A qualitative study, with an interpretative phenomenological approach with interviews	Community Healthcare Nurses (*n* = 10)	Perceptions on the construction of ethical competence.	Issues in the construction of the ethical competence: Personal values; Education; and Practice	6/10
Solum et al., 2016, Norway	To explore the challenges experienced by nurse teachers in Malawi in their efforts to enhance students’ moral competence in clinical practice.	A qualitative hermeneutic approach with interviews	Nurse teachers: individual interviews (*n* = 8) focus group interviews (*n* = 9)	Experiences of the challenges in enhancing student’s moral competence.	Overall themes: (1) authoritarian learning climate, with sub-themes (a) fear of making critical comments about clinical practice, (b) fear of disclosing mistakes and lack of knowledge, (c) lack of a culture of critical discussion and reflection that promotes moral competence; (2) discrepancy between expectations on learning outcome from nursing college and the learning opportunities in practice comprising three sub-themes (a) gap between the theory taught in class and learning opportunities in clinical practice, (b) lack of good role models, (c) lack of resources.	10/10
Zafarnia et al., 2017, Iran	To define and explain dimensions of moral competency among the clinical nurses of Iran.	A qualitative content analysis with in-depth semi-structured interviews and field notes	Clinical Nurses (*n* = 12)	Experiences of moral competence.	Main categories: (1) ‘moral character’, with sub-categories of altruism, search for meaning, be pioneering, perfectionism, self-control, honesty, and forgiveness; (2) ‘moral care’ with sub-categories of dignified care, safe care, fair care, and holistic care; (3) ‘moral decision-making’ with sub-categories of moral sensitivity, moral thinking, moral reasoning and moral courage.	8/10
**Cross-sectional studies**						Cross-sectional (eight items)
Asahara et al., 2014, Japan	The purpose of this study was to develop a valid and reliable moral competence self-assessment questionnaire for public health nurses that is easy to use in practice.	A quantitative descriptive, cross-sectional survey	Public Health Nurses (*n* = 3493)	Measurement of moral competence.	Moral Competence Questionnaire three factors with 15 items: Judgement based on the values of community members; Strong will to face difficult situations; Co-operating with relevant people/organisations.	5/8
Bužgová & Sikorová, 2013, Czech Republic	To determine the level of moral judgement competence in students of nursing, and whether it is influenced by the field of study, type of study, current year of study and age.	A cross-sectional survey	Nursing students (*n* = 662)	Measurement of moral judgement competence.	Moral judgement competence in nursing students showed mostly low and medium scores. On average, the students preferred stages 5 and 6 of moral judgement, that is the post-conventional level.	6/8
Göl & Arkan, 2022, Turkey	To determine the moral competence and intercultural sensitivity levels of nurses working in primary healthcare institutions and the relationship between the two.	A cross-sectional survey	Nurses (*n* = 83)	Measurement of moral competence.	The Intercultural Sensitivity Scale and Competence Questionnaire for Public Health Nurses-Turkish Version. Judgement based on the values of community members, strong will to face difficult situations, co-operating with the relevant people/organisations.	7/8
Katayama et al., 2022, Japan	To verify the reliability and validity of the Ethical Caring Competency Scale (ECCS) and to obtain suggestions for its use as an evaluation form in rubric format.	A descriptive and cross-sectional survey	Nurses (*n* = 962)	Measurement of ethical caring competence.	Core competencies: (1) Expressing the sensitivity and value of good care; (2) Acting while thinking about how to provide better care; (3) Creating indirect effects to provide better care; (4) Acting to learn what better care is.	6/8
Obeid & Man, 2020, Israel	To evaluate an advanced education workshop aimed at strengthening the self-perceptions of ethical competence by raising students’ self-efficacy when coping with ethical dilemmas.	A cross-sectional study	Nursing students (*n* = 127)	Educational intervention	Pedagogical axes: (1) The ethical axis – dealing with the principles of ethical thinking, values, and the guiding principles of ethical thinking; (2) The theoretical axis – theories that explore ethical dilemmas, a model for the resolution of ethical dilemmas; (3) The personal axis – emotional positions and a sense of the ability to cope with ethical dilemmas; (4) Professional experience – the encounter with an ethical issue that raises a dilemma.	5/8
Poikkeus et al., 2014, Finland	To analyse how nurse leaders support the ethical competence of nurses during recruitment and performance reviews.	A descriptive, cross-sectional survey	Nurse leaders (*n* = 198)	Support	Nurse leaders supported nurses’ ethical competence during performance reviews and recruitment. During recruitment, ethical behaviour and knowledge of nurses were ensured to varying degrees. During performance reviews, nurse leaders ensure that nurses meet the requirements for collegiality and comply with ethical guidelines according to nursing values and principles.	5/8
Poikkeus et al., 2018, Finland	To identify the level of nurses’ and nurse leaders’ ethical competence and analyse nurses’ and nurse leaders’ perceptions of support for nurses’ ethical competence at the organisational and individual levels and to examine associations between background factors and support.	A descriptive, cross-sectional survey	Nurses (*n* = 298)	Measurement	Ethical competence was estimated at an average level among nurses and at high level among nurse leaders and highest in ethical action and ethical reflection. High performance in acting according to laws and regulations, their organisation’s own values and principles, professional values as well as patients’ values. The ethical decision-making was estimated to be average among nurses, however, 90% estimated their ability to recognise ethical problems and ethical conflicts between different values on a high level.	8/8
			Nurse leaders (*n* = 193)			
Poikkeus et al., 2020, Finland	To examine relationships between nurses’ perceived organizational and individual support, ethical competence, ethical safety, and work satisfaction.	A cross-sectional questionnaire survey	Nurses (*n* = 298)	Support	Support generally low (44.5% and 38.3%, respectively). Moderate support: encouragement of ethical activity and support for compliance with laws and regulations (62% and 57%, respectively). Nurses’ overall perception of their ethical competence was moderate (68.8%).	8/8
van Schaik et al., 2021, The Netherlands	To evaluate the feasibility and first perceived outcomes of a newly developed clinical ethics support instrument called CURA.	A descriptive cross-sectional evaluation study	Nurses and certified nurse assistants (*n* = 97)	Support	The four steps of CURA intsrument are clearly described and easy to apply. The perceived outcomes showed that CURA helped respondents to reflect on moral challenges (71% strongly agreed), in perspective taking (67%), with being aware of moral challenges (63%) and in dealing with moral distress (54%). Organisational barriers: respondents could easily find time for using CURA (50% strongly agreed).	6/8
			Colleagues of course participants (*n* = 124)			
Vynckier et al., 2015, Belgium	To develop a valid and reliable instrument, named the ‘Students’ Perceived Effectiveness of Ethics Education Scale’ (SPEEES).	A quantitative descriptive non-experimental pilot study	3-year nursing students (*n* = 86)	Educational intervention	The content validity index/Ave scores for the subscales were 1.00, 1.00 and 0.86. The comprehensibility and user friendliness were favourable. Cronbach’s α was 0.94 for general effectiveness, 0.89 for teaching methods and 0.85 for ethical content. Students perceived case study, lecture and instructional dialogue to be effective teaching methods and general ethical concepts to contain effective content. Reflecting critically on their own values was mentioned as the only ethical competence that was promoted by the ethics courses.	6/8
Yildiz & Güdücü Tüfekci, 2017, Turkey	To adapt and evaluate the psychometric properties of the moral competence questionnaire for public health nurses in Turkey.	A methodological study	Public Health Nurses (*n* = 138)	Measurement	Three factors were extracted, which together explained a total of 67.50% of the variance. The Cronbach’s α values were .83, .91, .87, and .88 for factors 1, 2, and 3 and for the whole scale, respectively. Factors: (1) Strong will to face difficult situations, (2) Co-operating with relevant people/organizations, (3) Implementing the moral decision.	4/8
**Mixed-method studies**						Mixed-method (seven items) Comments
Kim et al., 2020, South Korea	To develop and evaluate a clinical ethics education program for nurses to improve their ethical confidence, ethical competence, and moral sensitivity.	A mixed-methods study	Registered nurses (*n* = 14)	Educational intervention of an ethics program.	The seven-session ethics program improved ethical confidence, ethical competence, and moral sensitivity in nurses. The Clinical Ethics Education Program included (1) sharing individual ethical issues in clinical settings; (2) understanding a process involved in ethical decision-making; (3) identifying ethical issues in end of-life care; (4) identifying ethical issues in family caregiving; (5) learning communication skills; (6) developing ethical leadership skills; (7) reflecting to build self-awareness of the significance of practicing clinical ethics.	2/7
		Focus group and questionnaire				Only purpose of the study was described, not the research questions
					Methods included (a) self-reflection; (b) lecture; (c) discussion; (d) film; e) video clip; (f) role play; (g) presentation	
**Quasi-experimental studies**						Quasi-experimental (nine items)
Chao et al., 2017, Taiwan	To develop and implement an interactive situational e-learning system, integrating nursing ethical decisions into a nursing ethics course, and to evaluate the effects of this course on student nurses’ ethical decision-making competence.	A quasi-experimental study, questionnaire	2-year nursing students (*n* = 100)	Educational intervention of a combination of traditional face to face teaching with an interactive multimedia online system.	Experimental group: significant improvement in nursing ethical decision-making competence, including skills in raising questions, recognizing differences, comparing differences, self-dialogue, taking action, and identifying the implications of decisions made, compared to their performance prior to the class. Students in the experimental group showed superiority to those in the control group in the competency of recognizing differences. The experimental course pushed students to search for and collect information needed to resolve the ethical dilemma.	7/9
			Experimental group (*n* = 51)			
			Control group (*n* = 49)			
Kälvemark Sporrong et al., 2007, Sweden	To evaluate the impact on perceived moral distress of an education and training program in ethics.	A controlled prospective study, questionnaire	Intervention department:	Educational intervention of three three-hour ethics lectures and three one-hour ethics rounds.	The Quality Work Competence (QWC)	8/9
			Nurses (*n* = 47)		Participants were positive about the training program. Moral distress did not change significantly. Lectures covered theories of ethics as a tool in ethical decision-making, theories of human dignity, and aspects of medical ethics such as prioritization in healthcare practice. The ethics rounds based on ethical situations in real-life workplace. Some of the participants prepared an authentic case before each round to be discussed and were moderated by the educators	
			Other (*n* = 10)			
			Control department:			
			Nurses (*n* = 101)			
			Other (*n* = 41)			
Martins et al., 2021, Portugal	To determine the influence of the bioethics teaching on the moral competence of medical and nursing students.	A longitudinal, descriptive study	Nursing students (*n* = 263)	Educational intervention as bioethics course	C-score was lower after the attendance of the ethics curricular units, with a statistically significant decrease for nursing students and not statistically significant for medical students. A multivariate analysis did not find any association between this decrease and gender, course or age.	6/9
			Medical students (*n* = 70)			
Martins et al., 2020, Portugal	To determine whether bioethics education in nursing influences the level of moral competence and opinion of nursing students about three ethical dilemmas.	A longitudinal, descriptive study	Nursing students (*n* = 122)	Educational intervention as bioethics course	Nursing students showed a moral competence stagnation, not statistically significant. Regarding performance for each of the dilemmas, students showed an increase in performance for the worker’s and judge’s dilemmas and a sharp decrease in performance for the doctor’s dilemma.	7/9
Tropec & Starcic, 2015, Slovenia	To conduct pedagogical experiment to identify the readiness and responsiveness of current organisation of nursing higher education in Slovenia.	A multiple-case quasi-experimental study	1-year nursing students:	Educational intervention as the course Philosophy and Professional Ethics in Nursing	No significant difference between the two learning settings.	8/9
			Case A (*n* = 40)		The students’ active engagement with the active learning methods in the group enables the development of ethical competences and the related communicative competences, interpersonal skills, collaboration and critical thinking.	
			Case B (*n* = 56)			
			Case C (*n* = 120)			

^a^Joanna Briggs Institute’s checklists and Mixed Method Appraisal Tool.

The designs of the studies were mainly qualitative^26,30–36,41,45,48,51^ or quantitative.^27–29,37,38,42–44,46,47,53^ One study had a mixed-method design,^50^ and a quasi-experimental design was used in five studies^25,39,40,49,52^ three of which had control groups.^25,49,52^ Data were mainly collected with interviews^26,30,31,33–36,41,45,48^ or questionnaires,^25,27–29,37–40,42–44,46,47,49,52,53^ one study used video filming,^51^ one used both questionnaire and focus group interview,^50^ and one used individual interviews and field notes.^32^

The participants were mainly nurses^29,32,34,37,38,41–43,50^ or nursing students.^30,35,40,44–47,49,51,52^ In addition, seven studies included a mix of healthcare professionals or students^25,26,28,31,39,48,53^ such as physicians or medical students; however, the majority of the participants in those studies were nurses or nursing students. Even though, in some studies the participants were nurse teachers^36^ or professors^33^ or nurse leaders,^27^ the articles focused on qualified nurses’ or nursing students’ moral competence and how to support it. The number of participants in qualitative studies ranged between 6 and 30, in quantitative studies between 83 and 3,493, and in quasi-experimental studies between 100 and 333 (Table 2). One mixed-method study included 14 participants.^50^

Third of the studies proposed that exploring stakeholder’s experiences,^30–32,36,41,45^ perceptions^34,48,51^ or views^26^ on competence was meaningful in order to describe and make visible the meaning and existence of competence. One study raised the promotion of moral competence on strategies and spaces as pedagogical teaching solutions.^33^ In several studies,^25,35,39,40,44,47,49,50,52^ the contributing factors were different educational interventions including basic education and continuing education. In addition, the interventions included ethics training programmes including ethics rounds, educational workshops, interactive e-learning interventions, pedagogical experiments and simulations. Six studies focused on the evaluation of the moral competence level of qualified nurses and nursing students.^28,37,38,42,43,46^ By operationalising moral competence, awareness of the required level becomes apparent, contributing to the promotion of moral competence. Finally, support from a third party, including superiors or organisational support structures such as clinical ethics support, was identified in three studies.^27,29,53^

### Factors contributing to the promotion of moral competence

**Figure 2.**
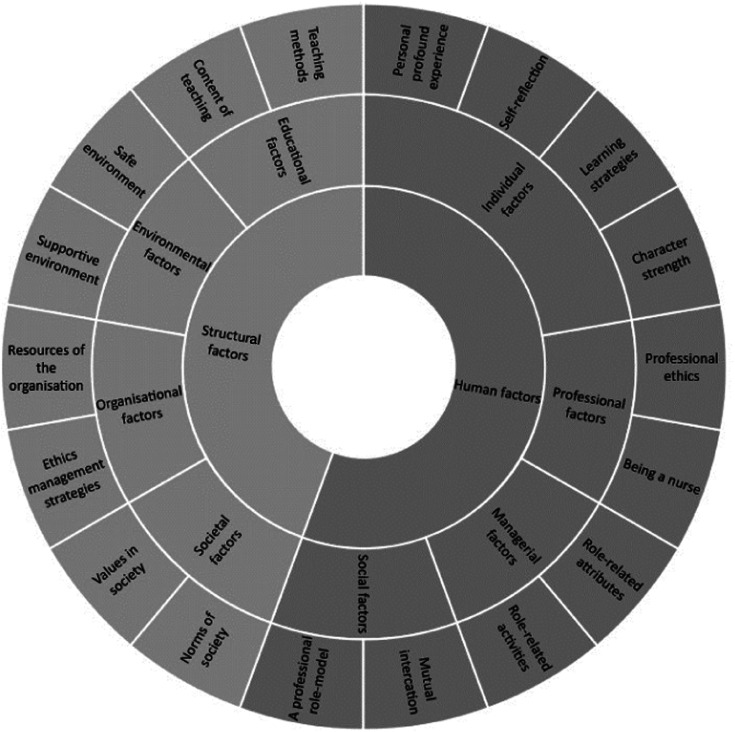
Moral competence contributing factors: Main categories, categories and sub-categories.

Factors contributing to the promotion of qualified nurses’ and nursing students’ moral competence were identified as comprising two main categories, human factors and structural factors, consisting of eight categories and eighteen sub-categories. Human factors consist of four categories: individual, social, managerial and professional factors, and ten sub-categories. Structural factors consist of four categories: educational, environmental, organisational and societal factors, and eight sub-categories. Human factors relate to the individual oneself, others such as patients, colleagues, managers and teachers and the nursing profession, whereas structural factors relate to the workplace (micro), organisation (meso) and society (macro) level structures (Figure 2, Table 3).

**Table 3.** Factors contributing to the promotion of moral competence.

Contributing factors	References
*Human factors*	
*Individual factors*	
Personal profound experience	Enderle et al. 2018,^1^ Kälvemark Sporrong 2007,^4^ Oddvang et al. 2021^1^
Character strength	Asahara et al. 2014,^2^ Borhani et al. 2010,^1^ Göl & Arkan 2022, Hemberg & Hemberg 2020,^1^ Peter et al. 2015,^1^ Ranjbar et al. 2018,^1^ Yildiz & Güdücü Tüfekci 2017,^2^ Zafarnia et al. 2017^1^
Self-reflection	Díaz Agea et al. 2018,^1^ Enderle et al. 2018,^1^ Katayama et al. 2022,^2^ Obeid & Man 2020,^2^ Oddvang et al. 2021,^1^ Peter et al. 2015,^1^ Schaefer & Junges 2014,^1^ van Schaik et al. 2021,^2^ Zafarnia et al. 2017^1^
Learning strategies	Bužgová & Sikorová, 2013,^2^ Díaz Agea et al. 2018,^1^ Katayama et al., 2022,^2^ Tropec & Starcic 2015^4^
*Social factors*	
A professional role model	Borhani et al. 2010,^1^ Maluwa et al. 2021,^1^ Ranjbar et al. 2018,^1^ Solum et al. 2016^1^
Mutual interaction	Asahara et al. 2014,^2^ Chao et al. 2017,^4^ Díaz Agea et al. 2018,^1^ Enderle et al. 2018,^1^ Göl & Arkan 2022,^2^ Kälvemark Sporrong 2007,^4^ Obeid & Man 2020,^2^ Poikkeus et al. 2020,^2^ Ranjbar et al. 2018,^1^ Yildiz & Güdücü Tüfekci 2017^2^
*Managerial factors*	
Role-related activities	Maluwa et al. 2021,^1^ Poikkeus et al. 2014,^2^ Poikkeus et al. 2018,^2^ Poikkeus et al. 2020^2^
Role-related attributes	Maluwa et al. 2021,^1^ Ranjbar et al. 2018^1^
*Professional factors*	
Being a nurse	Borhani et al. 2010,^1^ Hemberg & Hemberg 2020,^1^ Katayama et al. 2022,^2^ Kim et al. 2013,^3^ . Poikkeus et al. 2014,^2^ Ranjbar et al. 2018,^1^ Tropec & Starcic 2015,^4^ van Schaik et al. 2021^2^
Professional ethics	Poikkeus et al. 2020,^2^ Ranjbar et al. 2018^1^
*Structural factors*	
*Educational factors*	
Teaching methods	Borhani et al. 2010,^1^ Chao et al. 2017,^4^ Díaz Agea et al. 2018,^1^ Enderle et al. 2018,^1^ Kim et al. 2013,^3^ Kälvemark Sporrong 2007,^4^ Martins et al. 2021,^4^ Martins et al. 2020,^4^ Obeid & Man 2020,^2^ Oddvang et al. 2021,^1^ Schaefer & Junges 2014,^1^ Solum et al. 2016^1^ Tropec & Starcic 2015,^4^ van Schaik et al. 2021,^2^ Vynckier et al. 2015,^2^ Zafarnia et al. 2017^1^
Content of teaching	Chao et al. 2017,^4^ Díaz Agea et al. 2018,^1^ Enderle et al. 2018,^1^ Kim et al. 2013,^3^ Kälvemark Sporrong 2007,^4^ Martins et al. 2021,^4^ Martins et al. 2020,^4^ Obeid & Man 2020,^2^ Oddvang et al. 2021,^1^ Tropec & Starcic 2015,^4^ van Schaik et al. 2021,^2^ Vynckier et al. 2015^2^
*Environmental factors*	
Safe environment	Borhani et al. 2010,^1^ Díaz Agea et al. 2018,^1^ Solum et al. 2016^1^
Supportive environment	Borhani et al. 2010,^1^ Höglund et al. 2010,^1^ Poikkeus et al. 2018,^2^ Poikkeus et al. 2020^2^
*Organisational factors*	
Resources of the organisation	Maluwa et al. 2021,^1^ Poikkeus et al. 2018,^2^ Solum et al. 2016^1^
Ethics management strategies	Poikkeus et al. 2018,^2^ Poikkeus et al. 2020^2^
*Societal factors*	
Values in society	Asahara et al. 2014,^2^ Göl & Arkan 2022,^2^ Yildiz & Güdücü Tüfekci 2017^2^
Norms of society	Poikkeus et al. 2018,^2^ Poikkeus et al. 2020^2^

Study design.

Qualitative study.^1^

Quantitative study.^2^

Mixed-method study.^3^

Quasi-experimental study.^4^

#### Human factors

Individual factors contribute to the promotion of qualified nurses’ moral competence through the individuals themselves. This category comprises four sub-categories: **personal profound experience, character strength, self-reflection** and **learning strategies**. Personal profound experience means individuals having personal ethical experiences that they relate to practising as morally competent nurses.^25,33,35^ Character strength is about having a moral character with a desire and strong will to do good when managing difficult situations while providing nursing care.^26,30–32,37,42,43,45^ Self-reflection is an individual’s ability to reflect on the moral decision-making process and moral challenges and thinking while providing nursing care. Furthermore, it includes individuals recognising and reflecting on their own performance, ethical knowledge and personal values and exploring their own feelings.^32–35,38,45,47,51,53^ Learning strategies are comprised of an active role and engagement of the individual nurse in active learning.^38,46,49,51^

Social factors contribute to the promotion of qualified nurses’ moral competence through interaction and collaboration in workplace relationships. Two sub-categories were identified as **mutual interaction** and **a professional role model**. Mutual interaction takes different forms including discussions, argumentation and sharing experiences and knowledge. Furthermore, receiving support and feedback from educators and leaders as well as peer support are forms of mutual interaction.^25,29,31,33,37,42,43,47,51,52^ An individual’s moral competence develops by having a professional role model, such as manager, educator, peer or some other colleague who acts as an ethical example and whose practice, behaviour and moral characteristics are being observed and regarded as ethical.^30,31,36,48^

Two sub-categories of managerial factors were identified: **role-related attributes** and **role-related activities**. For contribution of moral competence, the manager should be humble, exemplary, approachable and flexible.^31,48^ In addition, they should possess knowledge on ethics and effective communication skills. Furthermore, managers with moral competence should demonstrate certain role-related activities such as providing feedback, rewarding ethical behaviour, supporting and encouraging ethical practice as well as arranging regular meetings to discuss ethical issues and performance and recruitment appraisals.^27–29,31^

Two sub-categories of professional factors were also identified as human factors: **professional ethics** and **being a nurse**. Professional ethics refers to the values and principles of the profession and nurses’ capacity to acknowledge, reason, commit and comply with them. Professional ethics pave the way for what it is to be a nurse.^29,31^ Being a nurse is understanding and using the ethical decision-making process from identifying and solving ethical problems to making decisions autonomously. It is about knowing one’s own responsibilities and collegiality as a nurse and aiming at better care for patients. In addition, being a nurse means expressing sensitivity and ability to deal with moral distress.^26,27,30,31,38,49,50,53^

#### Structural factors

Educational factors conducted by educational specialists contribute to the promotion of qualified nurses’ and nursing students’ moral competence through **teaching methods** and **content of teaching**, which were identified as two sub-categories. Traditional face-to-face teaching in a classroom as well as an interactive teaching using e-platforms as well as creative, problem-based and simulation-based teaching were methods of moral competence promotion.^25,30,32–36,39,40,44,47,49–53^ The foundation of teaching content are ethics theories, general ethical concepts as well as ethical principles and values. In addition, understanding the ethical decision-making process, from the identification of ethical issues and problems – whether hypothetical scenarios or realistic events – through ethical reasoning and reflection to ethical judgement and ethical practice, contributes to the promotion of qualified nurses’ and nursing students’ moral competence.^25,33,35,39,40,44,47,49–53^

Two sub-categories of environmental factors were identified: **safe environment** and **supportive environment**. A safe environment enables open reflection on ethical issues and values without the threat of negative responses.^30,36,51^ Furthermore, a supportive environment facilitating learning conditions and providing organisational structures encourages students and nurses to participate in ethical discussions and engage in ethical activity.^28–30,41^

Two sub-categories of organisational factors were also identified: **resources of the organisation** and **ethics management strategies**. Resources of the organisation comprise the availability of resources such as rules, guidelines and protocols. In addition, nurses from larger organisations reported having more ethical concerns/issues than those in smaller organisations where nurses felt more supported when addressing ethical issues.^28,36,48^ Ethics management strategies in the organisation are identified as the provision of information on ethical issues and enabling and arranging multidisciplinary discussions of ethical issues.^28,29^

Societal factors contribute to the promotion of qualified nurses’ moral competence by **values in society** and **norms of society**, which were the two sub-categories identified. Morally competent nurses should base their ethical judgement on the values of the community and comply with laws and regulations in their ethical decision-making. In addition, nurses considered that when the law and regulations were clarified, they had high competence in ethical decision-making.^28,29,37,42,43^

## Discussion

This review provides knowledge about the factors contributing to the promotion of qualified nurses’ and nursing students’ moral competence. This knowledge is useful for the development of ethics education interventions and everyday clinical practice. The results indicate that the moral competence can be promoted by various factors at all levels, from the level of an individual nurse or nursing student (micro) to society (macro) level. Given the importance of ethics in healthcare and the required moral competence of professionals,^4^ only a limited number of studies about factors contributing to the promotion of moral competence was discovered. However, the number of studies has been increasing during the past decade. Consistent with previous considerations,^3^ this indicates a growing interest in exploring nurses’ and nursing students’ moral competence. This can be explained by the increased requirement to prioritise scarce resources^6^ and address ethical issues^4^ and the need to alleviate the increased moral distress^54^ to which moral competence is a potential response.

The nature of the integrative method and the international character of the review allowed combining evidence produced with different study designs, identifying a broad perspective of the factors contributing to the promotion of moral competence. The studies, both qualitative and quantitative were mainly descriptive and based on nurses’ or nursing students’ experiences or their self-assessed level of moral competence, thus providing an insight into the contributing factors. In addition, some educational interventions were identified. However, in order to gain a deeper understanding and to see whether these factors are effective in contributing to the promotion of moral competence, more research is needed using intervention studies,^18^ implementation research and multiple methods designs.

Human and structural factors contributing to the promotion of moral competence were identified (Figure 2). Human factors were identified in relation to the individual oneself, others such as patients, colleagues, managers and teachers and the nursing profession, whereas structural factors were related to the workplace (micro), organisation (meso) and society (macro) level structures. Human factors that contribute to the promotion of moral competence relate mainly to informal practices and processes. This is supported by the literature stating that informal practices refer to socialisation processes, human encounters, interaction and professional autonomy.^55^ In addition, the findings indicate that structural factors create formal and structured practices, policies, strategies and programmes and enable informal opportunities for the systematic promotion of moral competence.^56^

The findings of human factors indicate that it is possible for individuals to promote their moral competence if they are empowered to practice as nurses and use their experiences in a positive way. In addition, it is expected that those who have chosen nursing as a career possess certain characteristics and are usually willing to help and do good to other people.^57^ In relation to other people, moral competence does not exist in a vacuum. Hence, the findings suggest that it is important for both nurses and nursing students to have ethical role models, colleagues and managers with whom to reflect critically on ethical issues^14,58^ in order to provide ethically high-quality care. In addition, it is stated that managers have a key role in promoting nurses’ moral competence by providing support and encouragement,^59^ which was also identified in this review.

The findings on structural factors indicate that attention must be paid to the content of teaching and the diversity of teaching methods when designing education and curricula.^12^ In addition, according to the findings, it is suggested that healthcare services need to build resilient organisations to support ethics management and enhance ethically sustainable nursing practices. Moral competence should also be given thorough consideration when making health policy decisions on a societal level. These may remove the impediments to nurses’ integrity and build psychological safety and a moral community where moral competence is effectively promoted.^4^ In addition, considering moral competence and the multiple levels of contributing factors,^10^ a complex intervention engaging stakeholders from the micro (patients, nurses, nursing students, managers and teachers) to the macro (policymakers) level is needed to effectively promote the moral competence of nurses and nursing students. Therefore, it is necessary to discuss whether the moral competence of individuals will broaden into ethical competence of the profession and the organisation. It is not enough to have morally competent individuals; highlighting professional ethics and supporting collective ethical competence as well as advancing value-based healthcare and care provision for patients is also needed.

### Limitations and strengths of the review

There are some limitations and strengths in this review. As a limitation, the literature search was performed by one researcher; however, the review protocol was registered in PROSPERO and followed throughout the process. The search strategy was developed among the research team and the search terms in collaboration with library informatics expert. In addition, preliminary searches were conducted by another person within the research team. Furthermore, reference lists of the included articles and relevant reviews were screened to identify all the relevant literature; however, it did not produce any new results.

A further limitation is that only studies written in English were included, introducing a potential selection bias. It should be noted that these studies originated from various countries, ensuring a multicultural perspective. Moreover, in order to ensure inclusiveness, no studies were excluded due to poor quality; as strengths any discrepancies were resolved among three researchers and according to the literature, excluding studies on the basis of poor quality is not recommended.^23^

As the final limitations, studies regarding the moral competence of mixed professionals were not excluded given that the majority of the participants were qualified nurses or nursing students. Furthermore, the data analysis process was performed by researchers from multiple countries, which may have introduced bias since only EU countries were included; however, no pre-defined frameworks were used, and the international perspective, as well as the inductive approach, may have prevented interpretation bias.

## Conclusion

Moral competence is a phenomenon that is essential to ensure ethically high-quality and sustainable healthcare. Research about the factors contributing to the promotion of qualified nurses’ and nursing students’ moral competence is limited but seems to be increasing. Human and structural factors were identified as the two main categories contributing to the promotion of moral competence from micro to macro levels. This review provides knowledge for researchers to develop interventions such as ethics education programs and to conduct implementation research. For nurse educators, this review provides knowledge to plan and develop ethics education, nursing education and nursing curricula. Moreover, this review provides knowledge for managers and organisations to create ethics structures that support and promote individuals’ moral competence, and for policymakers to enable the creation of such structures. It is important for both practice and education to pay attention to individuals’ ethical conduct by supporting and encouraging their moral competence and moral development from the beginning of basic nursing education throughout nursing careers and life-long learning. To gain a deeper understanding and to see whether the identified factors are effective in contributing to the promotion of moral competence, more research is needed using complex intervention, implementation and multiple methods designs, which would ensure ethically high-quality and sustainable healthcare.
